# Eyes on dementia: an overview of the interplay between eye movements and cognitive decline

**DOI:** 10.25122/jml-2023-0217

**Published:** 2023-05

**Authors:** Alec Ionescu, Emanuel Ştefănescu, Ştefan Strilciuc, Diana Alecsandra Grad, Dafin Mureşanu

**Affiliations:** 1. Department of Neuroscience, Iuliu Hatieganu University of Medicine and Pharmacy, Cluj-Napoca, Romania; 2. RoNeuro Institute for Neurological Research and Diagnostic, Cluj-Napoca, Romania; 3. Department of Public Health, Faculty of Political, Administrative and Communication Sciences, Babes-Bolyai University, Cluj-Napoca, Romania

**Keywords:** eye-tracking, dementia, eye movements

## Abstract

The economic and disease burden of dementia is forecasted to continue increasing. Considering its cognitive effects, timely diagnosis is important in developing a stage-based treatment plan and gathering data to support advocacy efforts and plan healthcare and social services. Eye-tracking technology has emerged as an efficient diagnostic tool in clinical practice and experimental studies. This review aimed to comprehensively analyze various aspects of eye-tracking technology, including pupillometry parameters, eye movements, eye-tracking devices, and neuropsychological tools. We conducted a systematic review retrieving articles published in the last ten years from six databases. Our results provide a complex overview for each included form of dementia/cognitive decline in terms of patient characteristics (age, sex-disaggregated by included pathologies), inclusion and exclusion criteria, devices, and neuropsychological tools. We also summarized findings on fixation stability tasks, saccadic evaluation, pupillometry, scene perception, object recognition, spatial memory, eye-tracking video tasks, and visual search. The eye-tracking method has become more common in cognitive assessments.

## INTRODUCTION

Cognitive decline, Alzheimer's disease, and other types of dementia pose significant challenges and represent a major public health issue, with recent estimates showing that 47 million individuals have dementia [1]. The global economic burden of dementia is estimated to reach US$818 billion by 2030 [2]. Dementia is a broad term that encompasses several types of cognitive disorders, including Alzheimer's disease (AD), vascular dementia (VaD), mixed Alzheimer's disease and vascular dementia (MAVD), Lewy body dementia (LBD), frontotemporal dementia (FTD), focal dementias, subcortical dementias, and secondary causes of dementia syndrome, such as intracranial lesions (ICLs) [3].

Alzheimer's disease is characterized by a gradual onset and incremental progression, with initial symptoms often characterized by short-term memory loss and unnoticed cognitive deficiencies. Conversely, vascular dementia is frequently linked to sudden vascular occurrences, and initial indications may include focal neurologic deficits. Lewy body disease (LBD) is characterized by a gradual onset and unpredictable advancement, accompanied by fluctuations in alertness and cognition. Focal atrophy in the frontal and/or anterior temporal lobes is a distinguishing characteristic of frontotemporal dementia alongside a gradual onset, with initial symptoms frequently manifesting as personality changes, reduced inhibitory control, speech impairment, and significant executive or language problems [4]. Considering the heterogeneity of dementia and its economic impact on society, timely diagnosis is of utter importance.

Eye-tracking technology has surfaced as a valuable and encouraging methodology for identifying and assessing cognitive dysfunction and monitoring its progression and severity [5]. Eye-tracking studies and the utilization of functional brain imaging techniques in the context of mental health and neurological disorder groups have substantially advanced our understanding of the impact of cognitive processes on saccadic eye movements [6]. The process of eye movement entails resolving the potential contradiction between top-down cognitive functions and bottom-up instinctive responses. This involves a conflict between the intentional exploration of our surroundings and the involuntary reaction to a visual or auditory stimulus that captures our attention. In an optimally functioning system, data travels from the visual cortex to the association cortex, followed by simultaneous and sequential projections to the premotor and motor cortex. There are also several back-and-forth connections between the cortex and the basal ganglia. The final collective output results from synaptic operations at multiple stages, generating a balance of information that either stimulates or suppresses neuronal activity. However, in cases of pathology, irregularities at varying stages could lead to distinctive patterns that have the potential to serve as a diagnostic indicator [7].

The main objective of this review article was to explore the application of eye-tracking technology in evaluating eye movements and pupillometry parameters across the spectrum of cognitive decline.

## MATERIAL AND METHODS

This review aimed to investigate the role of eye-tracking technology in evaluating eye movements and pupillometry parameters in different stages of cognitive decline. To achieve this aim, several specific objectives were addressed:

a. To provide an overview of the use of eye-tracking technology to evaluate eye movements and pupillometry parameters in different stages of cognitive decline, including its potential for early diagnosis and monitoring of cognitive decline.
b. To examine the different types of eye movements, including saccades, smooth pursuit, antisaccades, and pupillometry, and their relationship with cognitive decline.
c. To examine the role of eye-tracking technology in identifying early signs of cognitive decline and predicting dementia risk.
d. To map neuropsychological instruments and eye-tracking devices.

We conducted an extensive search using the following search strategy: “eye movements”, “eye tracking”, “cognitive dysfunction”, “aging”, and “dementia”. The employed search strategy was: ((“eye movement*” OR “saccade*” OR “smooth pursuit” OR “antisaccade*” OR “pupil*” OR “pupillometry” OR “fixation, ocular” OR “blink*”) AND (“cognitive dysfunction” OR “cognitive decline” OR “cognitive disorder” OR “cognitive impairment” OR “dementia” OR “Alzheimer's disease” OR “neurocognitive disorder”) AND (“eye tracking” OR “eye-tracking”)).

We queried six databases (PubMed, Web of Science, Scopus, PsycINFO, Cochrane Library, and Embase). We included only articles written in English and published between 2013 and 2023. Limitation regarding geographical coverage was not applied. An additional inclusion criterion encompassed possible aims/objectives for the included studies: investigating the relationship between eye movements and pupillometry parameters, as evaluated by eye-tracking technology, and cognitive dysfunction in aging and dementia.

Studies published before 2013, non-English articles, non-primary research articles, and studies that did not focus on the use of eye-tracking technology to evaluate eye movements and pupillometry parameters in assessing cognitive decline were excluded. Additionally, articles that primarily addressed ophthalmological disorders were also excluded.

Based on the inclusion/exclusion checklist, two reviewers screened the abstracts (first stage) and read the full-text articles in order to establish the eligibility (second stage) for the final (third) stage of retrieving the elements of interest. Across all stages, disagreements were resolved with a third reviewer.

The information extracted from the included articles was recorded in a Microsoft Excel workbook and included the following: title, country, year, first author, age data (by study arms, as mean, or, if the mean was not reported, as range), sample size (for each study group), sex (predominant group, number or percentage), recruitment site, diagnosis (definition), patient inclusion and exclusion criteria, eye tracking device, eye movement outcome measures, test protocol applied, instruments/scales used and corresponding scores (for each group or overall, for each instrument).

## RESULTS

Based on the search strategy, a total of 178 abstracts were retrieved. In the first stage, the abstracts were screened, resulting in 78 articles being included for full-text screening in the second stage. After the full-text screening, 35 articles were included in the review. Most studies were published in 2021 (n=7), while the least (n=2 for each year) were published in 2023, 2018, 2016, and 2013. The country with the highest number of studies was the United Kingdom (n=11).

As for the sample size, it ranged from 9 [8] to 108 for Alzheimer's disease [9], 7 and 20 patients for PCA [10, 11], 12 [12, 13] and 20 for bvFTD [14], and 15 [15] and 79 for mild cognitive impairment [16]. The highest and lowest mean age for patients with Alzheimer's disease was 77.8 [17] and 68.17 [18] years. For patients with posterior cortical atrophy (PCA), the mean ages ranged from 58.9 to 65.1 years [11, 19], and for patients with behavioral variant frontotemporal dementia (bvFTD), the mean age ranged from 62.2 [14] to 68.83 years [18]. In the analyzed studies, female participants were predominant in 20 studies, while male participants were predominant in 8 studies (for the other studies, a breakdown by sex was not included). Most patients were recruited in memory clinics or hospitals. Additional details for each study screened can be found in Table 1. Among the studies included in the analysis, seven lacked clearly defined inclusion criteria, while ten lacked exclusion criteria. In some studies, inclusion and diagnosis definitions overlapped. Additional details for each study screened can be found in Table 2.

**Table 1.** Participant characteristics and recruitment sites in the included studies

Author	Sample size (by study subgroups)	Participant's age (by mean or range)	Participants’ sex (by subgroups)	Recruitment sites
Sun *et al.* (2022)	AD, n=108; controls, n = 102	**Range overall:** 40 – 92 years	-	Cognitive impairment clinics
Laurens *et al.* (2019)	aMCI, n=25; mild AD, n=23; controls, n=26	-	-	Memory clinics (n = 3)
Shakespeare *et al.* (2015)	PCA, n=20; typical AD, n=17; controls, n=22	-	**Males:** PCA, n=8; typical AD, n=17; controls, n=5	-
Wilcockson *et al.* (2019)	Dementia due to AD, n=68; aMCI, n =42; Non-aMCI, n=47; controls, n=92	**Mean:** Dementia due to AD = 74, aMCI = 74, Non-aMCI = 69, Controls = 69	**Males:** Dementia due to AD = 50%, aMCI = 41%, Non-aMCI = 57%, Controls = 43%	Local memory clinics (National Health Service)
Chehrehnegar *et al.* (2019)	aMCI, n=40	**Mean:** AD = 73.52, aMCI = 68.1, Controls = 62.55	**Females:** AD, n = 14; aMCI, n=27; control, n=36	Brain and cognitive clinic
Zapoula *et al.* (2013)	MCI, n=15; AD, n=18; Controls, n=21	**Mean:** MCI=76; AD=76; Controls=73	**Females:** MCI, n=10; AD, n=14; Controls, n=12	-
Lage *et al.* (2021)	AD, n=18; bvFTD, n=18; svPPA, n=7; Controls, n=9	**Mean:** AD=68.17; bvFTD=68.83; svPPA=70.86; Controls=66.21	**Females:** AD, n=66.67%; bvFTD, n=22.22%; svPPA, n=57.14%; Controls, n=79.32%	Cognitive disorders unit
Crawford&Higham (2016)	Dementia, n=9; Controls, n=24	**Range:** Dementia=70-81 years; Controls=58-85 years	**Males:** dementia, n=8; Controls, n=13	Part of the lytham dementia study
Tadokoro *et al.* (2021)	MCI, n=52; AD, n=70; Controls, n=52	**Mean:** MCI=77.7; AD=77.8; Controls, n=76.7	**Females:** MCI, n=61.5%; AD, n=62.9%; Controls, n=63.5%	Hospital
Chehrehnegar *et al.* (2021)	aMCI, n=40; AD, n=21; Controls, n=59	**Mean:** aMCI=68.1; AD=73.52; Controls=62.55	**Females:** aMCI, n=27; AD, n=14; Controls, n=26	Memory clinic
Crawford *et al.* (2017)	Young, n=16; Older, n=15	**Range:** 18-30 (young); 50-77 (older)	-	-
Russell *et al.* (2021)	bvFTD, n=19; Controls, n=22	**Mean:** Controls = 64.2, BvFTD=63.7	**Females:** Controls, n=5, bvFTD, n=9	Longitudinal studies (Dementia Research Center)
Pavisic *et al.* (2021)	early PMCs, n=7; late PMCs, n=9; SMCs, n=9; Controls, n=26	**Mean:** early PMCs=38.1; late PMCs=41.3; SMCs, n=2; Controls, n=38.5	**Females:** early PMCs, n=5; late PMCs, n=6; SMCs=50; Controls=15	Dementia Research Center
Kim *et al.* (2022)	EOAD, n=19; LOAD, n=19; Controls =16	**Mean:** EOAD=64.5; LOAD=78; Controls =70	**Females:** EOAD, n=12 LOAD, n=12; Controls, n =7	Memory disorder clinic
Pavisic *et al.* (2017)	YOAD, n=36; Controls, n=21	**Mean:** YOAD=60.9; Controls=61	**Females:** YOAD=19; Controls=10	-
Primativo *et al.* (2017)	bvFTD, n=12; SD, n=6; Controls, n=38	**Mean:** bvFTD=67.7; SD=63.6; Controls=70.4	**Females:** bvFTD=2; SD=2; Controls=24	-
Opwonya *et al.* (2022)	MCI, n=79; Controls, n=170	**Mean:** MCI=73.3; Controls=71.5	**Females:** MCI=41, Controls=98	
Hutchings *et al.* (2018)	bvFTD, n=20; Controls, n=21	**Mean:** bvFTD=62.2; Controls=66.0	**Males:** bvFTD=11, Controls=11	Dementia research clinic
Russell *et al.* (2021)	bvFTD, n=18; Controls, n=22	**Mean:** bvFTD=63.9; Controls=64.2	**Males:** bvFTD=72%, Controls=59%	Research centre
Oyama *et al.* (2019)	MCI, n=26; dementia, n=27; Controls, n=27;	**Mean:** MCI=75.2, Dementia=71.5, Controls=27	**Males:** MCI, n=11; Dementia, n=11; Controls, n=9	Hospital
Fernandez&Parra (2021)	AD, n=18; Controls, n=18	AD=69; Controls=68	-	Hospital/clinic
El Haj *et al.* (2022)	AD, n=24; Controls, n=24	**Mean**: AD=72.33; Controls=70.96	**Females:** AD, n=14; Controls, n=13	Memory clinics
Shakespeare *et al.* (2015)	PCA, n=7; tAD (typical AD), n=8; Controls, n=19	**Mean:** PCA=58.9; tAD=69.7;	-	Research centre
Shakespeare *et al.* (2013)	PCA, n=13; Controls, n=10	**Mean:** PCA=65.1, Controls=63.1	**Males:** PCA, n=2, tAD, n=4, controls, n=5	-
Hannonen *et al.* (2022)	MCI, n=20 Mild AD dementia, n=21, Controls, n=37	**Mean:** MCI=72, Mild AD dementia=71, Controls=71	**Females:** MCI, n=9, Mild AD dementia, n=13, Controls, n=20	Brain research unit
Singleton *et al.* (2023)	bvAD, n=12; tAD, n=12, bvFTD,n=14; SCD, n=13	**Mean:** bvAD=66.6; tAD =64.6; bvFTD=66.4; SCD=57.5	**Males:** bvAD, n=75%; tAD, n=38.5%; bvFTD, n=64.3%; SCD, n=38.5%	Amsterdam Dementia cohort
Crawford *et al.* (2015)	AD, n=11; Controls, n=25	-	**Females:** AD, n=5; Controls, n=17	-
Polden *et al.* (2020)	AD, n=32; MCI, n=47	**Mean:** Alzheimer's disease=74.32; MCI=70.83	-	Sites and memory clinics (National Health System)
Xue *et al.* (2020)	SCD, n=14; aMCI, n=20; AD, n=15; Controls (young), n=34; Controls (old), n=30	**Mean:** SCD=67.6; aMCI=68.9; AD=70.1; Controls (young)=23; Controls (old)=65	**Females:** SCD, n=7; aMCI, n=12; AD=7; Controls (young), n=28; Controls (old), n=18	Neurology department
Pa *et al.* (2015)	n=43	**Mean:** 70.4	**Females:** n=22	Memory and aging center
McCade *et al.* (2018)	naMCI-md, n=18; aMCI-md , n=14; Controls, n=18	**Mean:** naMCI-md=63.78, aMCI-md=67.93, Controls=64.61	**Females:** naMCI-md, n=11; aMCI-md, n=9; Controls, n=11	Research institute
de Freitas Pereira *et al.* (2020)	MCI, n=51; mild dementia – AD, n=33; Controls = 43	Mean: MCI=68.33, mild dementia – AD = 72.97, Controls = 67.98	Females: MCI, n=41; mild dementia – AD, n=20; Controls, n=33	Memory clinic
Douglass *et al.* (2019)	bvFTD, n=15; Controls, n=17	-	-	Neuropsychiatry unit
Chau *et al.* (2016)	n=32	**Mean:** 77.9	**Females:** n=18	Outpatient memory clinic
Plaza-Rosales *et al.* (2023)	AD (early phase), n=9; controls, n=9	**Mean:** MCI=76.67, Controls=71.22	**Females:** MCI, n=7; Controls, n=5	Clinical hospital

AD - Alzheimer's disease, aMCI - Amnestic mild cognitive impairment, PCA - Posterior cortical atrophy, tAD - Typical Alzheimer's disease, bvFTD - Behavioral variant of frontotemporal dementia, svPPA - Semantic variant of primary progressive aphasia, MCI - Mild cognitive impairment, LOAD - Late-onset Alzheimer's disease, EOAD - Early-onset Alzheimer's disease, YOAD - Young-onset Alzheimer's disease, SD - Semantic dementia, SMCs - Symptomatic mutation carriers, PMCs - Presymptomatic mutation carriers, bvAD - Behavioral variant of Alzheimer's disease, SCD - Subjective cognitive decline, naMCI-md - Non-amnestic multiple domain mild cognitive impairment.

**Table 2.** Inclusion/exclusion criteria and definition of diagnosis

Author	Inclusion criteria	Exclusion criteria	Diagnosis definition
Sun *et al.* (2022)	-	“other neurological disease, uncorrected dysfunctions (for vision, hearing loss, aphasia), an inability to complete a clinical examination or scale assessment; history of mental disorders and illicit drug abuse; acute or chronic liver and kidney dysfunction, malignant tumors, other serious underlying diseases”	“clinical history, -neuropsychological examination, and structural imaging; other criteria (by the National Institute on Aging and the Alzheimer Association workgroup)”
Laurens *et al.* (2019)	“aMCI - prodromal AD research criteria“	“severe depression”	“mild AD – National Institute of Neurological and Communicative Disorders and Stroke and Alzheimer's Disease and Related Disorders Association (ADRDA) (MMSE score≥20)”
Shakespeare *et al.* (2015)	-	-	“PCA - standard clinical criteria; probable Alzheimer's disease –Dubois criteria for Alzheimer's disease”
Wilcockson *et al.* (2019)	“able to consent to study participation; capable to sign the informed consent”	“history of head trauma, stroke, cardiovascular disease, active or past alcohol or substance misuse or dependence, physical or mental condition severe enough to interfere with their ability to participate in the study; global or specific learning disability”	“dementia – clinical criteria for dementia due to AD, as per NINCDS-ADRDA criteria; MCI – subjective complaints of memory decline (patient or proxy); objective memory or other cognitive impairment with or without deficits in other cognitive domains; intact daily-life activities”
Chehrehnegar *et al.* (2019)	-	“other neurological or neuropsychiatric disorders, head trauma, substance abuse, medication use (affecting cognition)”	“aMCI –Petersen criteria (plus (MMSE)≥22 and Addenbrooke's Cognitive Examination (ACE)≥85; AD – Alzheimer disease criteria (Diagnostic and Statistical Manual of Mental Disorders)”
Zapoula *et al.* (2013)	“rating of MCI in seven domains (memory, attention, language, visual-spatial, orientation, calculation, and executive function), AD – fewer than two lacunar ischemia (of diameter <1 cm); scores of <4 (Hachinski Ischemia Scale), no history of significant systemic or psychiatric conditions or traumatic brain injuries (compromising brain function)”	“aMCI patients - impairment in a single non-memory domain (single, nonmemory domain MCI subtype) and impairment in two or more domains (multiple domains, slightly impaired MCI subtype)”	“Amnesia mild-cognitive impairment: memory complaint (supported by proxy), objective memory impairment, normal general cognitive function, intact ADL, absence of dementia; Petersen Mini-Mental State Examination (MMSE) cutoff score”
Lage *et al.* (2021)	“mild dementia stage (Global Deterioration Scale = 4); congruent neuropsychological and neuroimaging findings (brain CT and/or MRI), diagnoses confirmed by at least one type of biomarker, amyloid-PET, and/or CSF Alzheimer's disease core biomarkers; expert consensus (misclassification or heterogeneity)”	“no cognitive complaints and showed normal results in all baseline evaluations, including normal levels of CSF; a biomarker result discordant with their clinical group, 1 patient with a clinical diagnosis of probable Alzheimer's disease dementia due to normal levels of CSF biomarkers and negative PiB-PET; and two bvFTD patients and two svPPA patients due to positivity in PiB-PET”	“criteria for probable Alzheimer's disease, bvFTD, and svPPA; at least one type of Alzheimer's disease core biomarker”
Crawford&Higham (2016)	“probable Alzheimer's Disease (DSM IV), NINCDS criteria”	-	“probable Alzheimer's Disease (DSM IV), NINCDS criteria”
Tadokoro *et al.* (2021)	“MCI – mild cognitive decline in one or more cognitive domains, essentially preserved basic activities of daily living (ADL), the absence of dementia, delirium, or other mental disorders”	-	“Alzheimer’ disease (AD); mild cognitive impairment (MCI) - expert neurological clinicians”
Chehrehnegar *et al.* (2021)	“aMCI: Petersen criteria, (MMSE) ≥ 22; Addenbrooke's Cognitive Examination (ACE) ≥ 85 for aMCI group and (ACE) ≥ 78 for AD group; AD - (DSM-V) - psychiatrist or neurologist; review clinical history + physical examination”	“other neurological or neuropsychiatric disorder, depression, deficits in activities of daily living, head trauma, substance abuse, or using a medication that is known to affect cognition, ophthalmological diseases (glaucoma or macular degeneration), abnormal visual acuity (Snellen chart)”	“aMCI: Petersen criteria, (MMSE) ≥ 22; Addenbrooke's Cognitive Examination (ACE) ≥ 85 for a-MCI group and (ACE) ≥ 78 – AD group, AD (DSM-V) - psychiatrist or neurologist; review clinical history + physical examination”
Crawford *et al.* (2017)	“no psychiatric disorder, no psychoactive medication, no early signs of dementia, or general cognitive impairments (Mini-Mental State Examination); participants were screened for color blindness using the Ishihara Test (Ishihara, 1973), and for normal or corrected to normal visual acuity using a standardized Snellen chart”	-	“no psychiatric disorder (self-report), no psychoactive medication, no early signs of dementia, or general cognitive impairments (Mini-Mental State Examination) Participants were screened for color blindness using the Ishihara Test (Ishihara, 1973), and for normal or corrected to normal visual acuity using a standardized Snellen chart”
Russell *et al.* (2021)	“diagnostic criteria for bvFTD”	“diagnostic criteria for bvFTD”	“frontotemporal dementia - current diagnostic criteria for bvFTD were included in the study, of whom 10 were genetically confirmed (carrying mutations in chromosome 9 open reading frame 72 [C9orf72] = 5, progranulin [GRN] = 3 and microtubule associated protein tau [MAPT] = 2)”
Pavisic *et al.* (2021)	“autosomal dominant family history of AD and a known pathological mutation in PSEN1 or APP genes in at least one affected family member; Healthy individuals (without a family history of AD)”	-	“mutation analysis, estimated years to/from symptom onset (EYO), clinical assessment, a semi-structured interview, neurological examination, and the CDR scale, subjective cognitive decline questionnaires (MyCog, AD8)”
Kim *et al.* (2022)	“normal or corrected-to-normal visual acuity, more than 6 years of education, completion of a standardized neuropsychological battery (the Seoul Neuropsychological Screening Battery-SNSB); the Mini-Mental State Examination (MMSE) test; magnetic resonance imaging (MRI)”	“diseases that could affect cognitive function; moderate or severe vision loss (visual acuity <0.3) or a very low MMSE score (lower cutoff at 10) or an education level lower than the 6th grade were excluded from this study”	“AD - (NINCDS-ADRDA), MMSE score ≥ 10 and CDR; the age of onset - EOAD (first symptoms occurred between the ages of 45 and 65 years), LOAD (after the age of 65 years)”
Pavisic *et al.* (2017)	“standard criteria for PCA; AD - probable AD (National Institute of Aging clinical criteria)”	-	“PCA – standard criteria for PCA, AD – probable AD and fulfilled the NIA clinical criteria”
Primativo *et al.* (2017)	“consensus criteria for bvFTD, semantic dementia”	“bvFTD - pattern of deficits being better accounted for by other non-degenerative nervous system or medical disorders, behavioral disturbance being better accounted for by a psychiatric diagnosis, biomarkers strongly indicative of Alzheimer's disease or other neurodegenerative process, SD - both impaired confrontation naming and single-word comprehension, at least 3 of the following other diagnostic features must be present (impaired object knowledge, surface dyslexia or dysgraphia, spared repetition, spared speech production), imaging (predominant anterior temporal lobe atrophy), hypoperfusion or hypometabolism”	“bvFTD – a progressive deterioration of behavior and/or cognition by observation or history with three of the following symptoms being present (behavioral disinhibition, apathy, loss of sympathy or empathy, perseverative, stereotyped or compulsive/ritualistic behavior, hyperorality, and dietary changes, neuropsychological profile characterized by executive deficits with relative sparing of memory and visuospatial functions)”
Opwonya *et al.* (2022)	-	“difficult to measure due to color blindness, poor vision, or sagging eyelids, or because they did not pass the preliminary exercise and calibration test“	“MCI – Petersen criteria, CDR score of 0.5, neuropsychological test z scores were below -1.5 on at least one of five domain tests (according to age-, education-, and sex-specific norms)”
Hutchings *et al.* (2018)	-	“possible bvFTD, atypical presentation, history of psychiatric or neurological conditions, substance abuse or medication (affecting the central nervous system)”	“consensus following a clinical assessment with a behavioral neurologist, comprehensive neuropsychological assessment, structural brain imaging and met current consensus criteria for bvFTD”
Russell *et al.* (2021)	“diagnostic criteria – bvFTD”	-	“diagnostic criteria- bvFTD”
Oyama *et al.* (2019)	“MCI (revised Petersen criteria), dementia(DSM-IV)”	“no active neurologic or psychiatric diseases, with normal cognitive function, MMSE score between 25 and 30, and a CDR score of 0”	“MCI (revised Petersen criteria), dementia (DSM-IV)”
Fernandez & Parra (2021)	“at least one caregiver providing regular care and support, diagnosis of ophthalmologic diseases such as glaucoma, visually significant cataract, or macular degeneration”	“psychiatric diseases, traumatic brain injury, cardiovascular disease, brain tumors, or infectious diseases of the CNS; suffered from any medical conditions other than dementia that could account for, or interfere with, their cognitive functioning; evidence of vascular lesions in CT or MRI scans, evidence for an Axis I diagnosis (e.g., major depression or drug abuse) as defined by the DSM-IV”	“mild Alzheimer's Clinical Syndrome - (DSM-IV); subjects’ visual acuity was 20/20 or corrected to 20/20 as confirmed by an ophthalmological assessment”
El Haj *et al.* (2022)	“amnestic form of AD dementia –experienced neurologist or geriatrician (criteria of the National Institute on Aging-Alzheimer's Association)”	“significant psychiatric or neurological illnesses, alcohol or drug use, or a history of clinical depression, major visual or auditory acuity difficulties, administered drugs (e.g., tropicamide) - alter pupillary dilatation”	“amnestic form of AD dementia – experienced neurologist or geriatrician (criteria of the National Institute on Aging-Alzheimer's Association)”
Shakespeare *et al.* (2015)	“PCA - standard clinical criteria, had a clinical diagnosis of Alzheimer's disease and scored in the normal range (45th%ile) short Recognition Memory Test forwards “	-	“PCA - standard clinical criteria, had a clinical diagnosis of Alzheimer's disease and scored in the normal range (45th%ile) short Recognition Memory Test forwards“
Shakespeare *et al.* (2013)	“standard clinical criteria – PCA”	-	“standard clinical criteria – PCA”
Hannonen *et al.* (2022)	“AD dementia - revised National Institute on Aging and Alzheimer's Association criteria”	“diabetes, any signs of parkinsonism, upper motor neuron deficits, cerebellum disorders, dementia due to an etiology other than AD, moderate or severe AD (CDR 2 or 3)”	“AD dementia - revised National Institute on Aging and Alzheimer's Association (NIA/AA) criteria”
Singleton *et al.* (2023)	“at least two of six bvFTD features in conjunction with positive amyloid-β biomarkers based on CSF or PET examinations“	-	“proposed research criteria for this phenotype - ‘clinical bvAD’ as a combined behavioural and cognitive syndrome including two of five bvFTD behavioral features in conjunction with either memory or executive impairments, and defining additional levels (i.e., ‘possible’, ‘probable’ and ‘definite’ bvAD) based on different levels of biomarker, genetic and/or histological confirmation”
Crawford *et al.* (2015)	-	“a diagnosis of vascular or mixed dementia”	probable Alzheimer's - DSM IV and the National Institute of Neurological and Communicative Disorders and Stroke; Dementia severity - CDR”
Polden *et al.* (2020)	“AD – DSM-IV and the National Institute of Neurological and Communicative Disorders and Stroke (NINCDS); MCI - criteria, and a diagnosis of dementia due to mild cognitive impairment: subjective reports of memory decline (reported by the individual or caregiver/informant, memory and/or cognitive impairment (scores on standard cognitive tests were >1.5 SDs below age norms), activities of daily living were preserved”	“acute physical symptoms, focal cerebral lesions, history of neurological disease (e.g., Parkinson's disease, multiple sclerosis, epilepsy, amyotrophic lateral sclerosis, muscular dystrophy), cerebrovascular disorders (including ischemic stroke, haemorrhagic stroke, atherosclerosis), psychosis, active or past alcohol or substance misuse/dependence or any physical or mental condition severe enough to interfere with their ability to participate in the study”	“AD – DSM-IV and the National Institute of Neurological and Communicative Disorders and Stroke; MCI - criteria , and a diagnosis of dementia due to mild cognitive impairment: subjective reports of memory decline (reported by the individual or caregiver/informant), memory and/or cognitive impairment (scores on standard cognitive tests were >1.5 SDs below age norms), activities of daily living were preserved”
Xue *et al.* (2020)	“neuropsychological examination reports (done by clinicians)”	“CDR larger than 2 - moderate or severe AD status”	“CDR; conditions of subjective cognitive decline – SCD group, amnestic symptoms and the CDR of 0.5 – aMCI group, CDR of 1 – mild AD group”
Pa *et al.* (2015)	“CDR sum of boxes score of 0, a Mini-Mental State Examination score ≥ 28”	“poor data quality from excessive head motion; met criteria for mild cognitive impairment or dementia, neurological disorder that could affect cognition, significant psychiatric illness, head trauma with loss of consciousness greater than 10 minutes, severe sensory deficits, substance abuse, or were taking medications that affect cognition”	“CDR – functional abilities and the Neuropsychiatric Inventory (NPI) to evaluate behavior; Screening for depression – 30-item GDS”
McCade *et al.* (2018)	“multiple domain MCI - age ≥50 years; English as a first language; a MMSE score ≥24; intact basic facial processing abilities - Short Form Benton Facial Recognition Test (BFRT) score of >20”	“artifacts – interference from multifocal lenses and excessive blinking; psychiatric or neurological disorder (e.g., head injury, prior stroke, established dementia, intellectual disability, major depression, schizophrenia, substance abuse)”	“MCI - consensus of two neuropsychologists and one old Age Psychiatrist using established criteria, Individuals were also required to have preservation of function as evidenced by a Global Deterioration Scale score of ≤3; multiple-domain MCI – performance decrements <1.5 standard deviations (SDs) below age-based norms in at least two cognitive domains; aMCI-md clear evidence of memory storage (i.e., delayed recall), deficits on neuropsychological testing – impairments on at least one other cognitive domain, naMCI-md - deficits were present on multiple cognitive domains other than memory (e.g., processing speed, working memory, new learning, language, executive functioning)”
de Freitas Pereira *et al.* (2020)	“MCI – Mayo Clinic criteria, symptom severity (DSM-IV-R)”	“moderate or severe dementia, as well as those with non-AD dementia; neurological or psychiatric conditions/events, ocular diseases, moderate dementia or other type of dementia, calibration problems or low percentage of eye movement recordings”	“Alzheimer's disease (probable or possible) – DSM-IV-TR and the National Institute of Neurological and Communicative Disorders, Stroke-Alzheimer's Disease and Related Disorders Association criteria”
Douglass *et al.* (2019)	-	“history of stroke, alcoholism or substance abuse, bipolar disorder, major depressive disorder, learning disorder, schizophrenia, acquired brain injury, or any other neurological or psychiatric condition”	-
Chau *et al.* (2016)	“mild to moderate disease severity, no change in anti-dementia medications less than 1 month prior to study visit, no significant eye pathology, severe impairments in communication, or diagnosis of other neurological illnesses, including stroke during the two-year study period”	-	“Alzheimer's disease – criteria (DSM-IV-TR), (NINCDS-ADRDA)“
Plaza-Rosales *et al.* (2023)	“EEG signal quality”	“EEG – data quality issues; eAD group had evidence of non-degenerative dementia (e.g., inflammatory, metabolic, or vascular dementia), nonamnestic MCI or cognitive impairment of doubtful origin, or severe medical conditions that limited their ability to participate in the study”	“blinding process for patients’ performance - neurologist“

Our results indicate a high heterogeneity in the neuropsychological instruments used and the results obtained across the included articles. Thirty articles reported the type of neuropsychological instruments used, along with corresponding scores in some cases. The most commonly utilized neuropsychological instruments were the Mini-Mental State Examination (MMSE) (n=23), followed by the Clinical Dementia Rating (CDR) (n=8), Digit Span (n=7), Trail Making Test (TMT) and Geriatric Depression Scale (GDS) (n=5), Verbal fluency test (n=4), Graded Naming Test (GNT), Montreal Cognitive Assessment (MoCA), and Free and Cued Selective Reminding Test (FCSRT) (n=3) and Addenbrooke's Cognitive Examination III (ACE-III), Activities of Daily Living (ADL), British Picture Vocabulary Scale (BPVS), Delis-Kaplan Executive Function System (D-KEFS) Color-Word Inference test, Language, Memory, National Adult Reading Test (NART), Rey-Osterrieth Complex Figure Test (RVALT), Visuospatial, Wechsler Memory Scale-Revised (WMS-R), and Rey-Osterrieth Complex Figure Test/Ray's Copy (ROCFT/RCFT) (n=2) (Table 3).

**Table 3.** Neuropsychological instruments used and corresponding scores by study groups

Author	Instruments used	Instruments – scores (average by study group)
Laurens *et al.* (2019)	CDR (Clinical Dementia Rating), MMSE (Mini-Mental State Examination), DMS 48 (Delayed Matching-to-Sample Task 48) - immediate and delayed recall; FCSRT (Free and Cued Selective Reminding Test) - total recall and total delayed recall, Semantic verbal fluency, Letter verbal fluency, Trail Making Test A, Trail Making Test B, DSST (Digit Symbol Substitution Test), ADL+IADL (Independence in Activities of Daily Living), GDS (Geriatric Depression Scale)	**Controls:** CDR = 0, MMSE = 29, DMS 48 (immediate recall) = 47, DMS 48 (delayed recall) = 47, FCSRT (total recall) = 46, FCSRT (total delayed recall) = 16, Semantic verbal fluency = 32.2, Letter verbal fluency = 20.4, Trail Making Test A = 59.6, Trail Making Test B = 32.7, DSST = 58, ADL+IADL = 16.1, GDS = 3.9; **aMCI:** CDR = 0.5, MMSE = 26, DMS 48 (immediate recall) = 41, DMS 48 (delayed recall) = 42, FCSRT (total recall) = 30, FCSRT (total delayed recall) = 11, Semantic verbal fluency = 22.1, Letter verbal fluency = 18.2, Trail Making Test A = 47.3, Trail Making Test B = 19.2, DSST = 38, ADL+IADL = 14.3, GDS = 7.6; **AD:** CDR = 0.5, MMSE = 23, DMS 48 (immediate recall) = 39, DMS 48 (delayed recall) = 40, FCSRT (total recall) = 19, FCSRT (total delayed recall) = 6, Semantic verbal fluency = 19.2, Letter verbal fluency = 18.5, Trail Making Test A = 40.8, Trail Making Test B = 15.3, DSST = 32, ADL+IADL = 17.4, GDS = 5.4;
Wilcockson *et al.* (2019)	MoCA (Montreal Cognitive Assessment), FCSRT (free and total recall), Digit span total, Spatial total	**Controls:** MoCA total score = 28, FCSRT – Free Recall = 36.1, FCSRT – Total = 47.8, Digit span total =18.7, Spatial span total = 14.6; **Dementia due to AD:** MoCA total score = 20, FCSRT – Free Recall = 17.32, FCSRT – Total = 36.2, Digit span total =15.6, Spatial span total = 11.3; **Amnestic mild cognitive impairment:** MoCA total score = 21, FCSRT – Free Recall = 18.7, FCSRT – Total = 45.1, Digit span total =16.4, Spatial span total = 12.6; **Non-amnestic mild cognitive impairment:** MoCA total score = 25, FCSRT – Free Recall = 32.3, FCSRT – Total = 47.4, Digit span total =16.7, Spatial span total = 13;
Chehrehnegar *et al.* (2019)	MMSE, RVALT (Rey Auditory Verbal Learning Test), CDR, Barthel, GDS (Geriatric Depression Scale), ACE total score, Attention and orientation, Memory, Verbal fluency, Language, Visuospatial, Delayed memory	**Controls:** MMSE = 28.1, RVALT = 7.4, CDR = 0, Barthel = 99.57, GDS = 4.05, ACE total score = 90.01, Attention and orientation = 16.79, Memory = 12.47, Verbal fluency = 10.59, Language = 24.32, Visuospatial = 14.57, Delayed memory = 11.25 **aMCI:** MMSE = 25.62, RVALT = 5.73, CDR = 0.25, Barthel = 99.12, GDS = 6.2, ACE total score = 80.12, Attention and orientation = 15.22, Memory = 11.37, Verbal fluency = 9.2, Language = 20.42, Visuospatial = 13.52, Delayed memory = 10.37 **AD:** MMSE = 25.62, RVALT = 5.73, CDR = 0.25, Barthel = 99.12, GDS = 6.2, ACE total score = 80.12, Attention and orientation = 15.22, Memory = 11.37, Verbal fluency = 9.2, Language = 20.42, Visuospatial = 13.52, Delayed memory = 10.37
Zapoula *et al.* (2013)	MMSE, ADL	**Controls:** MMSE = 29, ADL = 15; **MCI:** MMSE = 26, ADL = 17; **AD:** MMSE = 16, ADL = 16
Lage *et al.* (2021)	MMSE, FCSRT Total Free and Cued Recall, FCSRT Delayed Free and Cued Recall, ROCFT Recall, ROCFT Copy, Imitative praxis, VOSP NL, Trail Making Test (A and B), Symbol digit test	**Controls:** MMSE = 28.96, FCSRT Total Free and Cued Recall = 42.96, FCSRT Delayed Free and Cued Recall = 15.07, ROCFT Recall = 16.04, ROCFT Copy = 32.86, Imitative praxis = 7.92, VOSP NL = 9.21, Trail Making Test A = 45.93, Trail Making Test B = 103.52, Symbol digit test = 39.55 **AD:** MMSE = 16.72, FCSRT Total Free and Cued Recall = 11.70, FCSRT Delayed Free and Cued Recall = 2.6, ROCFT Recall = 1.63, ROCFT Copy = 20.13, Imitative praxis = 6.40, VOSP NL = 6.50, Trail Making Test A = 157.10, Trail Making Test B = 179.50, Symbol digit test = 16.43 **BvFTD:** MMSE = 16.72, FCSRT Total Free and Cued Recall = 11.70, FCSRT Delayed Free and Cued Recall = 2.6, ROCFT Recall = 1.63, ROCFT Copy = 20.13, Imitative praxis = 6.40, VOSP NL = 6.50, Trail Making Test A = 157.10, Trail Making Test B = 179.50, Symbol digit test = 16.43 **SvPPA:** MMSE = 22.43, FCSRT Total Free and Cued Recall = 22, FCSRT Delayed Free and Cued Recall = 6, ROCFT Recall = 7.25, ROCFT Copy = 28.75, Imitative praxis = 8, VOSP NL = 8.80, Trail Making Test A = 84.80, Trail Making Test B = 145.50, Symbol digit test = 21.6
Tadokoro *et al.* (2021)	MMSE	**Controls:** 28.7; **MCI:** 27.2; **AD:** 20.1
Chehrehnegar *et al.* (2021)	MMSE, GDS, ADL, CDR, RVALT, Cognitive status, ACE (Addenbrooke's Cognitive Examination)	**Controls:** GDS = 4.05, ADL = 99.57, CDR = .09, MMSE = 28.16, RVALT = 7.40, ACE total score = 90.28, Attention and Orientation = 16.83, Memory = 12.47, Verbal fluency = 10.59, Language = 24.49, Visuospatial = 14.57, Delayed memory = 11.32; **aMCI:** GDS = 4.20, ADL = 99.12, CDR = 0.24, MMSE = 25.62, RVALT = 5.73, ACE total score = 80.20, Attention and Orientation = 15.22, Memory = 11.37, Verbal fluency = 9.2, Language = 20.42, Visuospatial = 13.52, Delayed memory = 10.45; **AD:** GDS = 3, ADL = 97.61, CDR = .45, MMSE = 22.04, RVALT = 4.22, ACE total score = 65, Attention and Orientation = 12.85, Memory = 8.42, Verbal fluency = 6.19, Language = 19.33, Visuospatial = 11.42, Delayed memory = 6.8;
Crawford *et al.* (2017)	Wechsler Adult Intelligence Scale III, Wechsler Memory Scale III, National Adult Reading Test, MMSE	-
Russell *et al.* (2021)	MMSE, CDR (incl. NACC FTLD), WMS-R (digit span backwards and forwards), D-KEFS Color-Word Interference Test, Trail Making Test A and B, BPVS (British Picture Vocabulary Scale)	**Controls:** MMSE = 29.5, CDR = 0.8. WMS-R Digit Span Forwards = 9, WMS-R Digit Span Backwards = 8.3, Phonemic Fluency = 15.1, D-KEFS Color-Word Interference Test = 56.5; Trail Making Test Part A (seconds) = 30.3, Part B = 69.2, British Picture Vocabulary Scale = 147.9 **bvFTD:** MMSE = 24.8, CDR = 10.3, WMS-R Digit Span Forwards = 6.8, WMS-R Digit Span Backwards = 4.8, Phoemic Fluency = 8.2, D-KEFS Color-Word Interference Test = 93.3; Trail Making Test Part A (seconds) = 52, Part B = 171.5, British Picture Vocabulary Scale = 124.9
Pavisic *et al.* (2021)	MMSE, NART (National Adult Reading Test), CDR (global), HADS-Anxiety, HADS- Depression, SCD:MyCog, AD8, Verbal IQ, Performance IQ, RMT (recognition memory test) - faces and words; Digit span (forwards and backwards), BPVS, Verbal fluency, Category fluency, GNT(Graded Naming Test), VOSP OD (Visual Object and Space Perception Battery), Stroop, Digit symbol, Camden PAL ( Camden paired associated learning), Spatial (forwards and backwards)	**Controls:** MMSE = 29.9, NART=29.7, CDR = 0, HADS-Anxiety=6.9, HADS-Depression=2, SCD: MyCog=1.5, AD8=0.5, Verbal IQ=101.6, Performance IQ=115.9, RMT faces =45.4, RMT words=48.9, Digit span forwards=7.2, Digit span backwards=4.9, BPVS=140.8, Verbal fluency=15.3, Category fluency=24.5, GNT/30=19.2, VOSP OD=18.6, Stroop ink time=48.4, Camden PAL=19.8, Digit symbol=65.9, Spatial forwards=6.4, Spatial backwards=5.8, Trails A=24.9, Trails B=54.2 **Early PMCs:** MMSE = 29.4, NART=26.9, CDR = 0, Anxiety=9, Depression=3.9, SCD: MyCog=5.1, AD8=0.5, Verbal IQ=102, Performance IQ=112.1, RMT faces =44.3, RMT words=50, Digit span forwards=6.9, Digit span backwards=4.9, BPVS=136.9, Verbal fluency=16, Category fluency=22.3, GNT/30=18.3, VOSP OD=17.7, Stroop ink time=51.4, Camden PAL=18.6, Digit symbol=65.6, Spatial forwards=5.4, Spatial backwards=4.9, Trails A=24.6, Trails B=58.1 **Late PMCs:** MMSE = 29.8, NART=30.9, CDR = 1.7, Anxiety=7, Depression=2, SCD: MyCog=3.6, AD8=0, Verbal IQ=105.9, Performance IQ=114.8, RMT faces =45.1, RMT words=47, Digit span forwards=7.3, Digit span backwards=5.2, BPVS=143.4, Verbal fluency=16.3, Category fluency=24.1, GNT/30=22.9, VOSP OD=19.1, Stroop ink time=48.3, Camden PAL=19.9, Digit symbol=66.7, Spatial forwards=5.9, Spatial backwards=5.1, Trails A=21, Trails B=46.3 **SMCs:** MMSE = 25, NART=30.9, CDR = 1.7, Anxiety=4.1, Depression=2.3, SCD: MyCog=15.9, AD8=5.3, Verbal IQ=97.2, Performance IQ=92, RMT faces =37.7, RMT words=34.4, Digit span forwards=6.2, Digit span backwards=4.3, BPVS=140.7, Verbal fluency=13.3, Category fluency=15.9, GNT/30=18.7, VOSP OD=17.1, Stroop ink time=99.3, Camden PAL=6.7, Digit symbol=31.1, Spatial forwards=4.1, Spatial backwards=3.4, Trails A=53.3, Trails B=153.6
Kim *et al.* (2022)	Digit span (forward and backward), K-BNT (Korean version of the Boston naming test), SVLT (immediate recall, delayed recall, recognition), RCFT (immediate recall, delayed recall, recognition), COWAT (animal, supermarket, phonemic), CDR, GDS	**Controls:** Digit span forward = 7, Digit span backward = 5, K-BNT=54, RCFT=35, SVLT – immediate recall = 24, SVLT delayed recall = 8, SVLT – recognition =23, RCFT – immediate recall = 21.5, RCFT – delayed recall=20, RCFT – recognition=21.5, COWAT animal=19, COWAT supermarket =19, COWAT phonemic=111, Stroop test color=30, MMSE=30, CDR=0.5, CDR (sum of box) = 0.5, GDS = 1.5 **EOAD:** Digit span forward = 6, Digit span backward = 3, K-BNT=44, RCFT=28, SVLT – immediate recall = 12, SVLT – delayed recall = 0, SVLT – recognition =15, RCFT – immediate recall = 2.5, RCFT – delayed recall=0, RCFT – recognition=16, COWAT animal=10, COWAT supermarket =9, COWAT phonemic=13, Stroop test color=46, MMSE=19, CDR=1, CDR (sum of box) = 5.5, GDS = 1.5 **LOAD:** Digit span forward = 6, Digit span backward = 3, K-BNT=29, RCFT=25, SVLT – immediate recall = 9, SVLT delayed recall=0 SVLT – recognition =15, RCFT – immediate recall = 0.5, RCFT – delayed recall=0, RCFT – recognition=15, COWAT animal=9, COWAT supermarket =9, COWAT phonemic=11, Stroop test color=25, MMSE=18, CDR=1, CDR (sum of box) = 7, GDS = 3.5
Pavisic *et al.* (2017)	MMSE, Visual acuity: Snellen, WASI (Wechsler Abbreviated Scale of Intelligence) - vocabulary and matrices, Digit Span (Forward and Backward), RMT (faces and words), GDA (Graded Difficulty Arithmetic)	**Controls:** MMSE=29.5, Visual acuity = NA, WASI (vocabulary) = 69, WASI (matrices) = 26.7, Digit Span Forward = 7.3, Digit Span Backward = 5.4, RMT (faces) = 24.7, RMT (words) = 24.4, GDA = 13.8 **YOAD:** MMSE=20.9, Visual acuity = 6/9, WASI (vocabulary) = 53.4, WASI (matrices) = 8.1, Digit Span Forward = 5.4, Digit Span Backward = 3.2, RMT (faces) = 19.5, RMT (words) = 17.5, GDA = 2.9
Primativo *et al.* (2017)	MMSE, WASI (matrices), WASI (vocabulary), Digit Span (forward), Digit Span (backward), Verbal fluency, Trails time, GDA (Graded Naming Test), Hayling Sentences, Brixton test	**Controls:** MMSE = NA, WASI (matrices) = 24.2, WASI (vocabulary) = NT, Digit Span (forward) = 9.1, Digit Span (backward) = 7.5, Verbal fluency = 16.7, Trails time = 49.1, Graded Naming Test = NT, Hayling Sentences = 6.4, Brixton test = 18.7 **SD:** MMSE = 26, WASI (matrices) = 25.5, WASI (vocabulary) = 41.5, Digit Span (forward) = 10.5, Digit Span (backward) = 9.5, Verbal fluency = 12.3, Trails time = 57.8, Graded Naming Test = 1.5, Hayling Sentences = 3.7, Brixton test = 22.5 **bvFTD:** MMSE = 25.1, WASI (matrices) = 17.1, WASI (vocabulary) = 44.8, Digit Span (forward) = 7.8, Digit Span (backward) = 5.5, Verbal fluency = 8.3, Trails time = 129.6, Graded Naming Test = 11.9, Hayling Sentences = 4, Brixton test = 28.3
Opwonya *et al.* (2022)	K-MMSE (Korean version of the Mini-Mental State Examination), Attention, Language, Visuospatial, Memory, Frontal	**Controls:** K-MMSE = 27.5, Attention = 9.8, Language = 0.2, Visuospatial = 0.6, Memory = 0.3, Frontal = 0.2; **MCI:** K-MMSE = 25.8, Attention = 8.3, Language = -0.1, Visuospatial = 0.2, Memory = -0.5, Frontal = -0.4
Hutchings *et al.* (2018)	ACE-III, Trails AB, RCF	**Controls:** ACE III (attention) = 17.1, ACE III (memory) = 24.5, ACE III (fluency) = 12.2, ACE III (language) = 25.7, ACE III (visuospatial) = 15.8, Digits forward = 7.2, Trails AB difference = 42.3, RCF – recall = 20.3 **bvFTD:** ACE III (attention) = 13.2, ACE III (memory) = 15.8, ACE III (fluency) = 6.3, ACE III (language) = 20.5, ACE III (visuospatial) = 13.8, Digits forward = 5.5, Trails AB difference = 123.6, RCF – recall = 7.5
Russell *et al.* (2021)	CDR (NACC FTLD), MMSE, WMS-R (Wechsler Memory Scale-Revised), Digit Span (backward and forward), Phonemic fluency, D-KEFS (Delis-Kaplan Executive Function System), Color-Word Inference Test, Trail Making Test (Part A and B), GNT, Mini-Social and Emotional Assessment	**Controls:** CDR (NACC FTLD) = 0.8, MMSE = 29.5, WMS-R Digit Span forward = 9, WMS-R Digit Span backward = 8.3, Phonemic fluency = 15.1, D-KEFS Color-Word Interference Test = 56.5, Trail Making Test part A = 30.3, Trail Making Test part B = 69.2, Graded Naming Test = 25.9, Mini-Social and Emotional Assessment Faux-Pas subtest = 12.9, Mini-Social and Emotional Assessment Faux-Pas subtest = 12.7; **bvFTD:** CDR (NACC FTLD) = 10.3, MMSE = 24.8, WMS-R Digit Span forward = 7, WMS-R Digit Span backward = 4.8, Phonemic fluency = 8.6, D-KEFS Color-Word Interference Test = 93.3, Trail Making Test part A = 51.7, Trail Making Test part B = 171.5, Graded Naming Test = 13.8, Mini-Social and Emotional Assessment Faux-Pas subtest = 10.2, Mini-Social and Emotional Assessment Faux-Pas subtest = 9.8
Oyama *et al.* (2019)	MMSE, FAB (Frontal Assessment Battery), ADAS (Alzheimer's Disease Assessment Scale), CDR	**Controls:** MMSE = 28.7, FAB = 13.6, ADAS-Cog = 4.4, CDR = 0 **MCI:** MMSE = 25.7, FAB = 13.4, ADAS-Cog = 9.4, CDR = 0.5 **Dementia:** MMSE = 16, FAB = 9.9, ADAS-Cog = 18.7, CDR = 1
Fernandez & Parra (2021)	MMSE, INECO (Institute of Cognitive Neurology) Frontal Screen, Trail Making Test A	**Control:** MMSE = 29.7, ACE-R = 98.5, INECO Frontal Screen = 29.3, Trial Making Test A = 35.8 **ACS:** MMSE = 23.1, ACE-R = 66.5, INECO Frontal Screen = 19.3, Trial Making Test A = 66.9
El Haj *et al.* (2022)	MMSE, WAIS-R	**AD:** MMSE=22.58
Shakespeare *et al.* (2013)	MMSE	Individual patient data
Hannonen *et al.* (2022)	CERAD (The Consortium to Establish a Registry for Alzheimer's Disease neuropsychological test battery), test battery, MMSE	**Controls:** verbal fluency = 25, naming = 13.4, MMSE = 28.4, Wordlist learning = 23.1, Wordlist delayed recall = 95.1, Wordlist recognition = 98.2, Visuo-construction = 10.5, Visuo-construction recall = 95.1, CERAD global memory score = 27.8 **MCI:** verbal fluency = 19.5, naming = 12, MMSE = 27, Wordlist learning = 17.8, Wordlist delayed recall = 75.5, Wordlist recognition = 85.3, Visuo-construction = 9.8, Visuo-construction recall = 85.1, CERAD global memory score = 23.1 **AD:** verbal fluency = 15.7, naming = 11.2, MMSE = 23.9, Wordlist learning = 13.1, Wordlist delayed recall = 38, Wordlist recognition = 75.3, Visuo-construction = 9.4, Visuo-construction recall = 58.9, CERAD global memory score = 17
Singleton *et al.* (2023)	MMSE, domain Z-score	**bvAD:** MMSE = 24.8, attention domain Z-score = -1.04, language domain Z-score=-1.22, memory domain Z-score=-2.34, executive domain Z-score=-1.49; **tAD:** MMSE = 24.7, attention domain Z-score = -0.94, language domain Z-score=-0.59, memory domain Z-score=-3.38, executive domain Z-score=-0.77; **bvFTD:** MMSE = 26.2, attention domain Z-score = -0.67, language domain Z-score=-1.5 memory domain Z-score=-1.36, executive domain Z-score=-1.11; **SCD:** MMSE = 28.1, attention domain Z-score = 0.17, language domain Z-score=0.05 memory domain Z-score=-0.14, executive domain Z-score=0.19;
Crawford *et al.* (2015)	MMSE, EADAS (Alzheimer's Disease Assessment Scale - European version)	**Control:** MMSE=29.3, EADAS=7.7 **AD:** MMSE=23.64, EADAS=19.91
Polden *et al.* (2020)	MoCA, Digit Span Task, Spatial Span Task	**AD:** MoCA = 20.19, Digit Span Task = 15.23, Spatial Span Task = 11.42; **MCI:** MoCA = 22.98, Digit Span Task = 15.95, Spatial Span Task = 12.93;
Pa *et al.* (2015)	GDS, MMSE, CDR, Modified Trails, Design Fluency, Stroop (Inhibition and Color Naming), Abstraction, Backward Digit Span	GDS = 2.9, MMSE = 29.6, CDR =0, Modified Trails Time = 24.4, Modified Trails Error=0.21, Stroop Inhibition = 51.3, Stroop Color Naming = 85.9, Abstraction = 5, Backward Digit Span = 5.3
McCade *et al.* (2018)	MMSE, WTAR (Wechsler Test of Adult Reading)-Predicted IQ, HAM-D (Hamilton Depression Rating Scale)	**Control:** MMSE=29.22, WTAR-Predicted IQ=105, HAM-D=2.06, Digit Span (SS) = 11.56, WMS-III LM I (SS) = 12.76, WMS-III LM II(SS) = 12.65, BNT (SS)=12.41, TMT-A(z-score) =0.53, COWAT(z-score)=0.61, TMT-B(z-score)=0.48 **aMCI-md:** MMSE=26.64, WTAR-Predicted IQ=103, HAM-D=3.36, Digit Span (SS) = 9.43, WMS-III LM I (SS) = 6.79, WMS-III LM II(SS) = 6.57, BNT (SS)=9.14, TMT-A(z-score) =-0.54, COWAT(z-score)=-0.14, TMT-B(z-score)=-2.28 **naMCI-md:** MMSE=28.61, WTAR-Predicted IQ=105.29, HAM-D=5.22, Digit Span (SS) = 10.28, WMS-III LM I (SS) = 9.17, WMS-III LM II(SS) = 9.83, BNT (SS)=10.29, TMT-A(z-score) =0.34, COWAT(z-score)=-0.17, TMT-B(z-score)=-0.54
de Freitas Pereira *et al.* (2020)	RAVLT, TMT, FDS, BDS, Rey (copy and recall), Verbal Fluency Test	**Control:** RAVLT total =50.45, RAVLT recall = 11, RAVLT recognition = 5.03, TMT-A= 39.74, TMT-B=84.03, FDS=8.73, BDS=6.43, Fluency=43.59, Rey copy=34.36, Rey recall=16.91 **MCI:** RAVLT total =42.55, RAVLT recall =8.28, RAVLT recognition = 2.55, TMT-A= 63.31, TMT-B=142.80, FDS=7.52, BDS=4.96, Fluency=36.78, Rey copy=33.02, Rey recall=15.42 **AD:** RAVLT total =26.35, RAVLT recall =2.73, RAVLT recognition = 0.64, TMT-A= 90.91, TMT-B=249.11, FDS=6.55, BDS=3.91, Fluency=24.55, Rey copy=25.48, Rey recall=7.55
Douglass *et al.* (2019)	NUCOG (Neuropsychiatry Cognitive Assessment too) - Attention, Spatial, Memory, Executive, Language	Individual patient data
Chau *et al.* (2016)	(standardized) MMSE, Conners's Continuous Performance Test Inattention	sMMSE=22.2, Conners's Continuous Performance Test Inattention=534.2
Plaza-Rosales *et al.* (2023)	CDR-SOB (Clinical Dementia Rating Scale Sum-of-Boxes), MoCA, MoCA-MIS (Montreal Cognitive Assessment Memory Index Score), MMSE	**MCI:** CDR-SOB=0.89, MoCA = 20.44, MoCA-MIS=9.56, MMSE =23.22 **Control:** CDR-SOB=0, MoCA = 29.22, MoCA-MIS=14.78, MMSE =29.78

The most frequently employed eye-tracking devices in the retrieved articles were different models of Tobii eye trackers (Pro spectrum system, TX300, ProX2-60, X120, TX300, 1750) and Eyelink (II, 1000, 1000 Plus). Prosaccade tasks were used in 9 studies, antisaccade tasks in 8 studies, visual search in 3 studies, fixation stability task and sinusoidal task, both in 2, while other applied protocols, such as Binding Task or Virtual Morris Water Navigation Task in one study (Table 4).

**Table 4.** Eye-tracking device, eye movement outcomes, and protocol

Author	Eye-tracking device	Eye movement outcome measures	Applied test protocol
Sun *et al.* (2022)	self-designed 3D eye-tracking system	fixation heatmaps	3D VPC task
Laurens *et al.* (2019)	Eyebrain T1® (EBT1) EyeBrain/Suricog® Society	error rates (wrong target)	spatial decision task
Shakespeare *et al.* (2015)	Eyelink II (SR Research)	number of square wave jerks, number of large intrusive saccades, longest period of fixation; time to first fixation upon target, amplitude, latency and velocity of first major saccade, number of saccades made; pursuit gain, number of saccades	fixation stability, saccade assessment (gap and overlap conditions), sinusoidal pursuit
Wilcockson *et al.* (2019)	EyeLink Desktop 1000 eye-tracker (SR Research)	antisaccade latency, antisaccade uncorrected errors	antisaccade task
Chehrehnegar *et al.* (2019)	SMI RED system (SensoMotoric Instruments)	first gain, latency, and velocity and final eye positions	prosaccade trial and antisaccade trial (GAP and OVERLAP)
Zapoula *et al.* (2013)	Eye See Cam	microsaccade rate (N/s), magnitude (deg) peak velocity (deg/s), duration (ms) intersaccadic interval (ms), direction (deviation from horizontal, deg) SWJ rate, percent of saccades in SWJs (%), SWJ magnitude (deg), SWJ direction (deviation from horizontal, deg)	20s fixation task
Lage *et al.* (2021)	OSCANN	parameters related to spatial accuracy, as saccade error (the deviation of the final position of the gaze from the target, measured as positive or negative error) and pursuit error (the difference between the target position and the gaze position during a pursuit test); parameters related to time, as latency (defined by the time delay between the appearance of a peripheral target and the onset of the ocular movement) and pursuit gain (the rate between ocular velocity and target velocity during a pursuit test); parameters related to success, as the percentage of correct memory saccades in the memory saccade test and, in the antisaccade test, the percentage of correct antisaccades, corrected erroneous antisaccades (corrected antisaccades) and successful antisaccades, which represent the sum of correct and corrected antisaccades	prosaccade task, sinusoidal smooth pursuit task, antisaccade task, memory saccade task
Crawford & Higham (2016)	‘ExpressEye’ (Optom, Freiburg, Germany)	saccade, antisaccade, the amplitude and reaction time of the primary saccade, proportion of correctly directed saccades (or errors) towards or away from the target, the amplitude, and latency of corrective saccades, the final eye positions	prosaccade task(PST), saccadic inhibition Go–No-Go tasks, antisaccade task(AST),
Tadokoro *et al.* (2021)	Gazefinder NP-100, JVC KENWOOD Corporation, Kanagawa, Japan)	eye tracking total scores (based on duration of fixations)	task videos during eye tracking
Chehrehnegar *et al.* (2021)	remote desktop eye tracker SMI RED system (SensoMotoric Instruments)	saccade reaction time (time to initiate saccades), saccade omission (fails to generate a saccade on a given trial), and number of anti-saccade errors uncorrected saccade	prosaccade task and antisaccade task (Gap and Overlap)
Crawford *et al.* (2017)	EyeLink II	saccade latencies, error rates and the spatial accuracy of saccades	prosaccade, antisaccade antisaccade – memory guided, antisaccade, Go/No-Go Condition
Russell *et al.* (2021)	Eyelink 1000 (and table-mounted eye-tracker)	fixation, small square wave jerk frequency, large square wave jerk frequency, number of large intrusive saccades, longest period of fixation, smooth pursuit, pursuit gain pro-saccades: amplitude error, saccade latency, peak velocity, anti-saccades, correct anti-saccades, self-corrected anti-saccades	fixation task, prosaccade task, antisaccade task, smooth pursuit task
Pavisic *et al.* (2021)	Eyelink	visual exploration strategies, total dwell time on fractals (ms)-‘DT’, equality score-‘Eq’, total shifts between fractals-‘S’, proportion of time spent on target-‘Pr’, basic oculomotor tasks, saccade amplitude (deg), saccade duration (ms), saccade velocity (deg/ms), peak velocity (deg/ms), number of saccades per second (sacc/s), blinks per trial	Object-localisation VSTM -task
Kim *et al.* (2022)	SMI Eye-Tracking Glasses 2 Wireless (SMI ETG 2w, SensoMotoric Instruments, Germany)	number and duration of fixations, number and duration of saccades, switching between two AOIs	semantic gaze mapping during videos
Pavisic *et al.* (2017)	Eyelink II (SR Research)	fixation stability: number of large intrusive saccades, number of square wave jerks, maximum fixation duration; pro-saccade: accuracy, time to fixate the target, number of saccades necessary to fixate the target; smooth pursuit task: pursuit gain, proportion of time pursuing the target	fixation stability task, smooth pursuit, prosaccade task
Primativo *et al.* (2017)	Eyelink II (SR Research)	basic oculomotor function - first saccade latency, time to fixate the first target, mean fixation duration, time to fixate the targets, total attempts of anticipatory saccades, correct anticipatory saccades, incorrect anticipatory saccades,	pursuit task (black dot moved across seven positions on the screen, following 12 different patterns)
Opwonya *et al.* (2022)	Tobii Pro spectrum system (Tobii Technology AB, Stockholm, Sweden)	invalid responses, correct responses, anticipatory errors, omissions, self-corrected- inhibition errors, uncorrected-inhibition errors	saccade responses - prosaccade, antisaccade, go-no-go tasks
Hutchings *et al.* (2018)	EyeLink 1000	number of fixations to regions of interest	Passively view faces appearing on the screen
Russell *et al.* (2021)	Eyelink 1000 Plus (SR research)	dwell time change score	pro-saccade task, simple emotion recognition task, complex emotion recognition task
Oyama *et al.* (2019)	Gazefinder NP-100 (JVC KENWOOD Corporation, Kanagawa, Japan)	% fixation duration within the ROI	task movies and pictures
Fernandez&Parra (2021)	EyeLink 1000 Desktop Mount (SR Research)	pupil size	Binding Task
El Haj *et al.* (2022)	eye-tracking glasses (Pupil Lab)	pupil size	span conditions (i.e., forward and backward), a control condition (i.e., counting)
Shakespeare *et al.* (2015)	Eye link II (SR Research, Canada)	saccade-gain task, fixation duration, saccade amplitude, central fixation bias	pro-saccade task Scene stimuli (30 photographic images)
Shakespeare *et al.* (2013)	Eye link II	duration of fixations, saccade amplitude proportion of fixations within ROI	free viewing paradigm
Hannonen *et al.* (2022)	Tobii TX300	total time to complete test (s), total number of errors, eye-tracking analysis, fixation duration (ms); saccade duration (ms); saccade amplitude (deg)	King-Devick reading test (ET)
Singleton *et al.* (2023)	Tobii ProX2-60	dwell time	Ekman 60 faces test (ET)
Crawford *et al.* (2015)	“ExpressEye” (Optom, Freiburg, Germany)	saccade Reaction time, saccadic amplitudes (degrees), saccadic direction (%correct)	gap prosaccade, overlap prosaccade, Go/No-Go Paradigm
Polden *et al.* (2020)	SR Eye Link Desktop 1000	gap effect (mean latency in the gap condition from the overlap condition mean latency)	prosaccade task (GAP and OVERLAP)
Xue *et al.* (2020)	Eyelink 1000 (SR Research Company, Canada)	interest-area first fixation duration, interest-area-fixation count	visual search performance task
Pa *et al.* (2015)	MRI-compatible infrared eye tracking system (Applied Sciences Laboratory Eye-Trac 6)	betweenness centrality total flow	prosaccade task, antisaccade task
McCade *et al.* (2018)	Tobii X120	mean fixation duration inside the ROI	visual processing task
de Freitas Pereira *et al.* (2020)	Tobii TX300	eye fixation and eye movement data; time to first fixation, fixations before (FB), fixation count (FC), duration of fixations (DF)	visual search task
Douglass *et al.* (2019)	Tobii 1750 eye tracker	accuracy, response time, fixation duration, number of fixations before a decision is reached, number of objects examined	visual search task
Chau *et al.* (2016)	visual attention scanning technology (VAST) (EL-MAR Inc., Toronto, Ontario, Canada)	average fixation duration, fixation frequency within images, relative fixation time	visual attention task
Plaza-Rosales *et al.* (2023)	Eyelink 1000	blinks, fixations, and saccades	Virtual Morris Water Navigation (VMWN) task

Tasks aimed at assessing fixation stability yield quantifiable measurements, such as the number of square wave jerks, small square wave jerk frequency, large square wave jerk frequency, maximum fixation duration, or the longest fixation period [10, 20, 21].

The findings from fixation stability paradigms reveal distinct patterns of impairment across different forms of dementia. Decreased periods of fixation have been observed in both the PCA and typical Alzheimer's disease groups when compared to the control. Intriguingly, in PCA, this decrease in the fixation period was accompanied by an elevated frequency of large intrusive saccades, whereas typical Alzheimer's disease was linked to an increased frequency of square wave jerks [10]. Behavioral variant frontotemporal dementia was associated with impaired fixation stability compared to control participants [21]. Furthermore, individuals with young-onset Alzheimer's disease (YOAD) exhibited an increased frequency of large intrusive saccades in conjunction with reduced fixation duration while performing the fixation stability task. Patients with YOAD displayed a significant negative correlation between their performance in object decision, fragmented letters, and dot-counting tests and the presence of large intrusive saccades [20].

One study revealed that 80% of patients with posterior cortical atrophy (PCA) exhibited oculomotor impairment in prosaccade tasks, which was in stark contrast to the 17% of patients with typical Alzheimer's disease and the 5% of control participants [10]. Notably, a considerable distinction was found between the groups of individuals with PCA and those with typical Alzheimer's disease regarding saccade amplitude error, with a sensitivity of 93.8% and specificity of 83.3% [10]. Interestingly, in amnestic mild cognitive impairment (aMCI) patients, the gap and overlap prosaccade paradigm showed impaired saccadic gains, making it a sensitive measure for distinguishing between aMCI and healthy controls [22]. There was no significant difference in pro-saccade task performance between patients with bvFTD and controls except for the vertical overlap saccadic task [21]. For individuals with young-onset Alzheimer's disease, impaired performance was observed in the pro-saccade task, characterized by reduced accuracy, longer fixation times, and greater saccadic movements required to fixate the target. Furthermore, these metrics negatively correlated with performance on several neuropsychological tests [20]. Lastly, patients with mild cognitive impairment (MCI) were found to have significantly longer latencies in prosaccade tasks compared to healthy controls [23].

The antisaccade task demonstrated alterations in the process of healthy aging and the initial phases of neurodegeneration. Key hubs in the oculomotor network, particularly the right lateral nodes linked to the right lateral frontal eye field (rlatFEF), have been identified as critical for efficient executive functioning during aging. Dysfunction in these hubs and network connections may be a potential biomarker for cognitive decline [24]. The process of aging is associated with alterations in both inhibitory control (IC) and working memory (WM), as further demonstrated by an increased antisaccade error rate [25]. This behavior is also observed in individuals diagnosed with amnestic mild cognitive impairment. These patients exhibit more errors and omissions and make fewer corrections in their saccade behavior compared to controls [26].

Furthermore, patients with MCI showed a lower proportion of correct responses and an increased number of inhibition errors in both PS/AS and Go/No-go tasks. In addition, patients with MCI showed a trend toward increased correction latencies [16]. The antisaccade task presented the potential to differentiate between aMCI and non-amnestic MCI (naMCI) [27]. Distinct oculomotor patterns have been observed in patients with Alzheimer's disease, behavioral variant frontotemporal dementia, and semantic variant primary progressive aphasia (svPPA). BvFTD patients, in particular, exhibit significant deficits in the antisaccade and memory saccade tasks, which heavily rely on frontal lobe functioning and require cognitive demand [18]. These oculomotor patterns involve the dorsolateral prefrontal and parietal cortices, as well as the striatum [28].

In a longitudinal assessment with eye tracking, patients with AD initially had slower reaction times than the control group. However, after 12 months, both groups displayed similar reductions in reaction times to the gap stimulus compared to the overlap stimulus. Moreover, there was a general improvement for both groups in the accuracy of saccades and reaction time speed after 12 months [29].

Individuals diagnosed with AD or mild cognitive impairment had a higher number of oblique microsaccades than individuals without these conditions [30].

Pupillometry studies have shown significant potential in assessing cognitive disorders. For example, in a binding task, healthy controls exhibited significant pupil dilation during the Bound Colours condition compared to the Unbound Colours condition. However, this differentiation was not observed in individuals with Alzheimer's clinical syndrome. These aberrant pupil responses effectively differentiated Alzheimer's clinical syndrome patients from healthy controls with 100% sensitivity and specificity [31]. Interestingly, in another study focused on assessing pupil size during complex cognitive tasks such as forward spans, backward spans, and counting, patients with AD showed fewer variations in pupil size across the conditions compared to the control participants [32].

Regarding scene perception abilities, the eye-tracking evaluation showed that patients with posterior cortical atrophy struggle to focus on task-relevant regions, highlighting the interplay between cognition and perception [33]. People recall object locations better than their identities using the change detection eye tracking model, implying stronger visual working memory for real-world scenes than object recognition. Interestingly, this capacity to process and remember visual-spatial information in naturalistic settings persists in individuals with mild cognitive impairment, indicating that their condition does not hinder this aspect of cognition [34]. In a visual memory task, presymptomatic carriers of familial Alzheimer's disease (FAD) showed increased reliance on fixation time for target localization. Whereas only symptomatic individuals showed memory function deficits, indicating potential spatial memory issues in presymptomatic FAD carriers [35].

Eye-tracking scores, calculated from fixation duration during a video observation task, significantly decreased in MCI and AD, correlating strongly with MMSE scores. The test effectively differentiated between NC, MCI, and AD, particularly in memory and reasoning tasks [36]. Similarly, another eye-tracking video task showed that patients with AD exhibited an increased number of fixations and longer fixation duration in perceptual and working spaces than normal control (NC) participants. This was especially evident in patients with early-onset AD (EOAD), who had more fixations and higher switching than both late-onset AD (LOAD) patients and NC participants [37]. Video-based eye-tracking tasks also showed excellent diagnostic accuracy in distinguishing MCI subjects from healthy controls, comparable to the MMSE scores [38].

In a visual search eye-tracking task based on fixation parameters, patient groups displayed higher interest area fixation count than controls, with a pronounced disparity between AD participants and the aMCI or subjective cognitive decline (SCD) group [39]. Similarly, patients with MCI and AD showed increased screen fixations and longer fixation durations during target searches compared to controls, often focusing more on distractors. Machine learning techniques were able to effectively differentiate between control participants and those with AD [40]. In the case of bvFTD, visual search patterns were characterized by reduced accuracy, longer response times, and an elevated occurrence of eye movements, both in terms of quantity and duration [41].

Other complex eye-tracking tasks have proven effective in studying the visual behavior of cognitively impaired patients. One such method involves reading studies using eye-tracking techniques. For example, an eye-tracking adapted version of the King-Devick reading test observed notable distinctions in saccadic duration and amplitude between control individuals and those with MCI or AD dementia [42]. In addition to reading tasks, eye-tracking has been employed in visual-spatial decision-making tasks. In one study, patients with AD displayed higher error rates than individuals with aMCI and the control group [43]. The utility of eye-tracking extends even further to complex tasks such as visual attention paradigms. For instance, in a study examining visual attention scanning in Alzheimer's disease, patients who spent less time viewing new images presented a higher reduction in neuropsychological evaluation scores [44]. This suggests that the inclination towards novelty, evaluated through eye-tracking technology, could be a potential marker for disease progression and cognitive decline [44]. Furthermore, individuals with aMCI exhibited difficulties in spatial learning, as demonstrated in an eye-tracking adapted Morris Water navigation task [8]. Interestingly, eye-tracking data obtained from a 3D Visual Paired Comparisons (VPC) task revealed distinct differences in eye-tracking traits between people with Alzheimer's Disease (PwAD) and healthy controls (HCs), as evident from the fixation heatmaps [45].

## DISCUSSION

Our review provides a comprehensive overview of eye-tracking technologies, eye movements, and neuropsychological instruments used in patients with different stages of cognitive decline. Most studies included cohorts of patients who have Alzheimer's Disease. A recent systematic analysis investigating the global burden of AD and other types of dementia revealed significant increases in incidence and prevalence rates between 1990 and 2019. The analysis reported a significant increase of 147.95% in incidence and 160.84% in prevalence. Furthermore, the number of deaths attributed to dementia increased by 1.06 million during this period [46]. Most cases of AD and dementia were reported among women, and the burden of these conditions was higher in high-income countries [46].

The Mini-Mental State Examination (MMSE) was the most commonly used scale in the retrieved studies [10, 33]. The MMSE is a cognitive screening tool consisting of 11 questions that assess various cognitive domains such as orientation, attention/concentration, memory, language skills, and visuospatial abilities [47]. It has been extensively validated in multiple languages and has been utilized in patients diagnosed with various pathologies, including Parkinson's disease, Alzheimer's disease, traumatic brain injury, and depression, as well as in different clinical settings such as clinical practice, clinical trials, and epidemiological studies [48–50]. However, some challenges related to acceptability, ease of scoring, and the influence of factors like age, education, language, and culture have been identified [51].

The second most used instrument, the Clinical Dementia Rating (CDR), is utilized for assessing the stages of dementia. It evaluates various domains of interest, including memory, orientation, judgment and problem-solving, community affairs, home and hobbies, and personal care. The CDR assigns five ratings, ranging from “Healthy CDR 0” to “Severe Dementia CDR 3.0”, to indicate the severity of dementia. Although this instrument has demonstrated good inter-reliability, it has a difficult scoring system [52]. Nevertheless, the CDR has been validated in culturally diverse populations and is widely employed in clinical practice and clinical trials [53–56].

The Trail Making Test (TMT) was the third most commonly used assessment tool, followed by Digit Span and the Geriatric Depression Scale (GDS). The TMT is a neuropsychological test that evaluates memory and executive functioning by measuring the time taken to connect consecutive circles on a page (TMT-A) and to switch between numbers and letters (TMT-B). Average and deficient scores for TMT-A range from 29 to 78 seconds, and for TMT-B, 75 to 273 seconds [57]. This test, from which other versions have been derived, is available in both electronic and paper-based versions and has been validated in multiple countries and different age segments [58–62]. Digit Span, constructed on the work of Gottfried Leibniz on cognition, is one the most used subtests to assess short and working memory by repeating a row of digits forward and backward [63, 64]. It is incorporated in the WAIS-IV (Wechsler Adult Intelligence Scales) and has been validated by taking into account different indicators, such as “age-corrected scaled scores” or “various time-to-recite measures” [65,66]. GDS is a 30-item binary self-reported measure assessing the affective and cognitive domains for signs of depression (higher scores corresponding to severe cases of depression) [67]. It has been extensively used in various populations, including hospitalized and non-hospitalized elderly individuals with cancer, traumatic brain injury, and stroke. The scale has been translated and validated in multiple languages, and a shorter 15-item version (GDS-S) has been derived to reduce respondent fatigue [68].

Our extensive literature review identified various types of devices used in eye-tracking protocols. These devices ranged from self-designed 3D eye-tracking systems to commercially available devices, such as EyeLink II, EyeLink 1000, and EyeLink 1000 Plus [31, 33, 35, 39, 45, 69–73]. Tobii devices, such as Tobii Tx300, Tobii ProX2-60, Tobii X120, 1750, and Tobii Pro Spectrum, were also frequently employed by researchers [34, 41, 42, 74–77]. Other notable devices include the SMI Red system, Eye Brain T1, OSCANN, Gazefinder NP-100, ExpressEye, Eye-Trac6, and Visual Attention Scanning Technology (VAST) [22, 24, 30, 37, 38, 44, 78–82]. Other studies used eye-tracking glasses such as SMI Eye-Tracking Glasses 2 and Pupil Lab [32, 37]. Even web camera-based systems like Eye See Cam have been used in eye-tracking studies [30]. The availability and diversity of these devices provide researchers with a wide range of options to investigate and better understand cognitive dysfunction.

Diverse eye-tracking protocols have been employed to evaluate various oculomotor functions and visual processing abilities in individuals experiencing cognitive dysfunction. The review provides an overview of various tasks that are frequently utilized in research studies. These tasks encompass the 3D VPC task, spatial decision task, fixation stability assessment, saccade assessment under gap and overlap conditions, sinusoidal pursuit tasks, antisaccade tasks, memory-guided saccade tasks, and object-localization VSTM tasks [10, 20–22, 24, 30, 33, 35, 45, 69, 71, 73, 78, 80–84]. Furthermore, the integration of eye-tracking technology with video stimuli has facilitated the investigation of various cognitive processes, including emotion recognition, passive viewing of faces and scene stimuli, visual search, visual attention, visual processing, and reading tasks such as virtual Morris Water Navigation [36, 37, 39, 41, 44, 75, 75, 76]. This review highlights the wide range of eye-tracking protocols available, which offer researchers a thorough understanding of how to investigate cognitive dysfunction related to dementia. Through the utilization of these protocols, researchers can augment their comprehension of the pathophysiology of dementia and potentially make valuable contributions to advancing more efficacious diagnostic and therapeutic methodologies.

Fixation stability tasks include the evaluation of gaze fixation stability, wherein various parameters such as square wave jerks, fixation duration, and saccade frequencies are measured. Research has indicated that various manifestations of dementia correlate with compromised fixation stability [10,20]. Both PCA and typical Alzheimer's disease are characterized by a reduction in fixation duration. However, PCA is characterized by a higher occurrence of large saccades, whereas AD is associated with an increased prevalence of square wave jerks [10]. Impaired fixation stability has been observed in individuals diagnosed with bvFTD[41]. Individuals diagnosed with YOAD exhibit a heightened occurrence of large saccades and a decrease in the duration of fixation, both of which are inversely associated with cognitive test performance [20]. Research studies have demonstrated that individuals diagnosed with PCA exhibit deficits in their ability to perform saccadic evaluation tasks, indicating impairments in their oculomotor function [10]. Similarly, patients diagnosed with amnestic mild cognitive impairment display compromised saccadic gains, further highlighting the impact of cognitive impairment on oculomotor abilities [22]. Antisaccade tasks have been employed as a means of distinguishing between cognitive disorders, specifically amnestic mild cognitive impairment (aMCI) and non-amnestic mild cognitive impairment (naMCI)[22]. Research on pupillometry has demonstrated promise in distinguishing individuals with AD from those without the condition. [32]. Eye-tracking has been employed to evaluate various cognitive tasks in individuals with dementia, including scene perception, object recognition, spatial memory, video tasks, and visual search [34, 72, 81, 85]. This method has unveiled unique eye movement patterns and holds promise as a diagnostic tool.

## OCNCLUSION

Our review mapped the role of eye-tracking technology in evaluating eye movements and pupillometry parameters, combined with several scales, across different stages of cognitive decline associated with various forms of dementia. Although we included the most recent articles, a constant update is needed to account for increasing trends of dementia, healthcare digitization, and combinations of eye-tracking methodologies.
